# Non-Native Ambrosia Beetles as Opportunistic Exploiters of Living but Weakened Trees

**DOI:** 10.1371/journal.pone.0131496

**Published:** 2015-07-02

**Authors:** Christopher M. Ranger, Peter B. Schultz, Steven D. Frank, Juang H. Chong, Michael E. Reding

**Affiliations:** 1 USDA Agricultural Research Service, Horticultural Insects Research Lab, and Department of Entomology, Ohio Agricultural Research and Development Center, The Ohio State University, Wooster, Ohio, United States of America; 2 Hampton Roads Agricultural Research and Extension Center, Virginia Polytechnic Institute and State University, Virginia Beach, Virginia, United States of America; 3 Department of Entomology, North Carolina State University, Raleigh, North Carolina, United States of America; 4 Pee Dee Research and Education Center, Clemson University, Florence, South Carolina, United States of America; University of Wisconsin-Madison, UNITED STATES

## Abstract

Exotic *Xylosandrus* spp. ambrosia beetles established in non-native habitats have been associated with sudden and extensive attacks on a diverse range of living trees, but factors driving their shift from dying/dead hosts to living and healthy ones are not well understood. We sought to characterize the role of host physiological condition on preference and colonization by two invaders, *Xylosandrus germanus* and *Xylosandrus crassiusculus*. When given free-choice under field conditions among flooded and non-flooded deciduous tree species of varying intolerance to flooding, beetles attacked flood-intolerant tree species over more tolerant species within 3 days of initiating flood stress. In particular, flood-intolerant flowering dogwood (*Cornus florida*) sustained more attacks than flood-tolerant species, including silver maple (*Acer saccharinum*) and swamp white oak (*Quercus bicolor*). Ethanol, a key host-derived attractant, was detected at higher concentrations 3 days after initiating flooding within stems of flood intolerant species compared to tolerant and non-flooded species. A positive correlation was also detected between ethanol concentrations in stem tissue and cumulative ambrosia beetle attacks. When adult *X*. *germanus* and *X*. *crassiusculus* were confined with no-choice to stems of flood-stressed and non-flooded *C*. *florida*, more ejected sawdust resulting from tunneling activity was associated with the flood-stressed trees. Furthermore, living foundresses, eggs, larvae, and pupae were only detected within galleries created in stems of flood-stressed trees. Despite a capability to attack diverse tree genera, *X*. *germanus* and *X*. *crassiusculus* efficiently distinguished among varying host qualities and preferentially targeted trees based on their intolerance of flood stress. Non-flooded trees were not preferred or successfully colonized. This study demonstrates the host-selection strategy exhibited by *X*. *germanus* and *X*. *crassiusculus* in non-native habitats involves detection of stress-induced ethanol emission and early colonization of living but weakened trees.

## Introduction

Non-native ambrosia beetles (Coleoptera: Curculionidae) in the tribe Xyleborini have been regularly intercepted at ports of entry and several species are now established and abundant in North America [1–9]. A cryptic nature, diet of symbiotic fungi, sib-mating, haplodiploidy, broad host range, and interactions with naïve hosts likely aid in their introduction and establishment in non-native habitats [1–9]. In particular, two species native to Southeast Asia, *Xylosandrus germanus* (Blandford) and *Xylosandrus crassiusculus* (Motschulsky), have become widely established across regions of North America [2,10]. As of 2010, *X*. *germanus* and *X*. *crassiusculus* were found in 32 and 29 of 50 states, respectively [10]. *Xylosandrus germanus* has also become established throughout much of Europe [11–16]. *Xylosandrus crassiusculus* currently has a narrower distribution in Europe, but is established in Central America and the Caribbean, East and West Africa, and Oceania [17,18].

*Xylosandrus germanus* and *X*. *crassiusculus* exhibit a capability to attack a diverse range of >200 and >120 species, respectively [19,20], and have extensively attacked living and apparently-healthy trees growing within non-native ornamental, horticultural, and forested settings [9,11,12,21–26]. However, the basis for these attacks is not well understood, particularly since both species colonize dying and dead trees in their native habitats [4]. Over a dozen additional species of ambrosia beetles that normally attack dying or dead trees have also shifted to attacking living trees in non-native habitats; thereby leading to speculation about the mechanisms underlying their selection of living trees as hosts [4,9,27].

As with other Scolytinae, host-derived olfactory cues, particularly ethanol, play an important role during host selection by *X*. *germanus* and *X*. *crassiusculus* [9,28–30]. Ethanol is a key host-derived attractant and baiting, irrigating, or injecting trees with ethanol induced attacks on specific trees [9,28,31–33]. *Xylosandrus germanus* also efficiently located and attacked ethanol-injected trees, but rarely landed on and did not attack adjacent trees not emitting ethanol [9]. A positive correlation was also demonstrated between concentration of ethanol injected into a tree and cumulative ambrosia beetle attacks [31], along with a positive correlation between emission rates from lures and corresponding trap captures [29]. In addition to using host-derived volatiles, some Scolytinae and Platypodinae ambrosia beetles produce aggregation pheromones to attract conspecifics and overwhelm tree defenses [34,35]. However, this strategy is uncommon among the Xyleborini ambrosia beetles and *X*. *germanus* and *X*. *crassiusculus* are not anticipated to produce aggregation pheromones [9].

Ethanol is rapidly induced by trees in response to a variety of abiotic and biotic stressors, including flood and drought stress, girdling, freezing, pathogens, root and crown disturbance, and pollutants [36–43]. In the case of flooding, roots subjected to little or no oxygen will switch from aerobic to anaerobic cellular respiration [41,42]. Pyruvate formed during glycolysis is then converted into ethanol and either metabolized by alcohol dehydrogenase or emitted from the epidermis. Field and experimental observations have documented the potential for flood stress to predispose trees to attack by non-native ambrosia beetles. For instance, extensive ambrosia beetle attacks were detected in May 2011 on field grown dogwood (*Cornus florida* × *Cornus kousa*) trees that were subjected to water logging and poor soil drainage at two ornamental nurseries following record setting precipitation [43]. Subsequent experiments demonstrated flood-stressed *C*. *florida*, which is considered intolerant of flooding, were more attractive and preferentially attacked by *X*. *germanus* and *X*. *crassiusculus* [43]. Ethanol was also detected within the vascular tissue and emitted from the epidermis of *C*. *florida*, but not from non-flooded trees.

Comparatively high concentrations of ethanol can be associated with flood intolerant trees, but some tolerant species delay or avoid accumulating ethanol [44,45]. Thus, allowing *X*. *germanus* and *X*. *crassiusculus* to choose among tree species varying in their tolerance of flood stress could provide insight into their preference for trees in a specific physiological condition. Previous studies indirectly suggested *X*. *germanus* could distinguish among even slight differences in tree quality [9,21,22]. Furthermore, the extent to which *X*. *germanus* and *X*. *crassiusculus* utilize healthy naïve trees in non-native habitats, especially in the absence of weakened ones, is also poorly understood and unsubstantiated. Attacks have been suspected to occur on apparently-healthy trees [10,21,46–48], but such trees may actually have been inapparently-stressed at the time of attack [9]. Comparing colonization success on flood-stressed and non-flooded trees could thereby provide crucial insight into host utilization within non-native habitats and aid in characterizing factors contributing to the invasiveness of *X*. *germanus* and *X*. *crassiusculus*.

The overall goal of this study was to assess factors involved with *X*. *germanus* and *X*. *crassiusculus* targeting living trees in non-native habitats, particularly the role of host physiological condition on preference behavior and colonization success. To characterize host preference specificity, we tested if tree species intolerant of flooding would be preferentially attacked under free choice conditions over species more tolerant of flooding. Ethanol in stem tissues of non-flooded and flood-stressed trees was also analyzed to determine if elevated concentrations were associated with the more intolerant species. To elucidate limitations imposed by tree condition on colonization success, we also compared the capability of *X*. *germanus* and *X*. *crassiusculus* to colonize flood-stressed and non-flooded trees. We hypothesized that *X*. *germanus* and *X*. *crassiusculus* can distinguish among trees of varying physiological conditions and preferentially attack species intolerant of flood stress, but exhibit non-preference and poor colonization of non-flooded trees. We also hypothesized interspecific variability in ethanol stem concentrations would be related to intolerance of flood stress.

## Materials and Methods

### Preference among Trees Varying in their Tolerance of Flood Stress

Free-choice experiments were conducted in Ohio and Virginia to characterize the role of host physiological condition on preference of *X*. *germanus* and *X*. *crassiusculus* and to determine if tree species intolerant of flooding would be preferentially attacked over moderately tolerant or tolerant species. Flowering dogwood *C*. *florida* (≈20 mm stem diam. at 15.24 cm above soil), Japanese snowbell *Styrax japonicus* S. et Z. (≈23 mm), and eastern redbud *Cercis canadensis* L. (≈25 mm) were selected as representatives of flood-stress intolerant species [49–51]; flowering cherry *Prunus serrulata* Lindl. (≈28 mm), American elm *Ulmus americana* L. (≈19 mm), and river birch *Betula nigra* L. (≈21 mm), were selected as moderately tolerant species [49,52,53]; and swamp white oak *Quercus bicolor* Willd. (≈19 mm) and silver maple *Acer saccharinum* L. (≈19 mm) were selected as tolerant species for deployment in Ohio [49,51]. *Cornus florida* and *C*. *canadensis* were selected as intolerant species; *P*. *serrulata* and *M*. *virginiana* represented moderately tolerant species; and *Q*. *bicolor* and *A*. *saccharinum* represented tolerant species for deployment in Virginia. Trees were 2−3 years old and maintained in 19-L pots.

Flooding conditions were imposed using a pot-in-pot system according to Ranger et al. [43], whereby a 26-L pot was first lined with a plastic waste bag of 3 mil (0.076 mm) thickness. A 19-L pot containing a single tree was then placed within the plastic lined pot. Flood stress was initiated by irrigating the media within the internal pot until standing water pooled around the base of the trees. Trees were checked daily to ensure standing water was maintained in the selected pots throughout the duration of experiments. Excess plastic liner surrounding the edge of the flooded pot was draped around the internal circumference of the pot and wrapped around the base of the stem to prevent beetles from landing in the standing water.

Non-flooded and flooded specimens of *A*. *saccharinum*, *B*. *nigra*, *C*. *canadensis*, *C*. *florida*, *P*. *serrulata*, *Q*. *bicolor*, *S*. *japonicus*, and *U*. *americana* were deployed in eight randomized complete blocks within a woodlot in Ohio (40°45’41.97”N 81°51’20.89”W). Permission was provided by The Ohio State University to use the described field sites. There was approximately 3 m between trees within each block and 10 m between adjacent blocks. Flood stress was initiated on 29 April 2013 and maintained until 14 May 2013. Similarly, non-flooded and flooded specimens of *A*. *saccharinum*, *C*. *canadensis*, *C*. *florida*, *M*. *virginiana*, *P*. *serrulata*, and *Q*. *bicolor* and were deployed in six randomized complete blocks along the edge of a woodlot in Virginia (37°17’17.64”N 76°39’0.07”W). Permission was provided by the Department of Parks and Recreation, York County, Virginia. Flood stress was initiated on 8 April 2013 and maintained until 23 April 2013 in Virginia. Trees were inspected for ambrosia beetle attacks one day after initiating flood stress and then every two to three days until the experiment was terminated.

Trees deployed in Ohio that sustained at least one ambrosia beetle attack were cut at the roots under field conditions on the last day of the experiment and subsequently transferred to a walk-in refrigerator held at 5°C. Trees were then dissected to recover foundress ambrosia beetles from the tunnels and galleries, and specimens were preserved in 95% ethanol for identification. Attacked trees deployed in Virginia were also cut at the base on the last day of the experiment and stem sections were held in plastic containers at room temperature until beetles were excavated for identification. No protected species were sampled during the course of this study.

### Association of Ethanol with Trees Varying in their Tolerance of Flood Stress

Ethanol was quantified within the vascular tissue of the flood-stressed and non-flooded trees deployed in Ohio and Virginia as part of the experiment evaluating ambrosia beetle preference for trees varying in their tolerance of flooding. Based on Ranger et al. [43], three days after initiating flooding an Osborne arch punch (C.S. Osborne & Co., Harrison, New Jersey) was used to take four tissue core samples (1 mm depth, 5 mm diam.) at 10 cm above the base from flooded and non-flooded specimens of *A*. *saccharinum*, *B*. *nigra*, *C*. *canadensis*, *C*. *florida*, *P*. *serrulata*, *Q*. *bicolor*, *S*. *japonicus*, and *U*. *americana* deployed in Ohio. Similarly, tissue samples were also taken three days after initiating flooding from flood-stressed and non-flooded specimens of *A*. *saccharinum*, *C*. *canadensis*, *C*. *florida*, *P*. *serrulata*, *Q*. *bicolor*, and *M*. *virginiana* deployed in Virginia. The superficial tissue core samples included the outer bark, phloem, and vascular cambium, but not the sapwood and heartwood. Tissue core samples were placed in 2 mL Eppendorf tubes immediately after sampling, which were then covered among dry ice until transported back to the laboratory. Tissue samples were stored at -80°C until analysis. Tissues samples taken in Virginia were packed with dry ice and shipped overnight to the USDA-ARS-HIRL for analysis.

Solid phase microextraction-gas chromatography-mass spectrometry (SPME-GC-MS) was used to analyze ethanol content [9,43]. In short, phloem and vascular cambium tissues from the four core samples were placed in 2 mL glass vials and suspended in a water bath at 100°C for 30 min. Vials were removed from the water bath and a SPME fiber was exposed to the headspace within the vial for 5 min. The fiber was retracted after sampling and the syringe was immediately capped with a sealed section of PTFE microbore tubing (0.568 mm inner diam. × 1.07 mm outer diam., Cole-Parmer, Vernon Hills, Illinois) to prevent contamination of the fiber. A coating of carboxen-polydimethylsiloxane (CAR/PDMS; 75 μm coating; Sigma-Aldrich, St. Louis, Missouri) was used, which is ideal for gases and low molecular weight compounds (MW 30−225) [54].

Immediately after sampling, syringes were inserted into the injection port of a GC (Varian CP-3800; Varian Inc., Palo Alto, California) equipped with a Merlin Microseal septum system (Sigma-Aldrich) and thermally desorbed for 2 min at 250°C. The injection port was operated in splitless mode from 0–2 min and then split at a ratio of 1:20 for the remainder of the run. A capillary nonpolar DB-5 column (0.25 μm × 30 m × 0.25 μm; cross-linked/surface bonded 5% phenyl, 95% methylpolysiloxane; Agilent J&W, Santa Clara, California) was used for analysis according to the following program: 40–60°C at 3°C/min and 60–230°C at 20°C/min. A Varian 2200 MS detector was operated in electron impact mode with a scan range of 14−415 *m/z*. System control was accomplished with Star Chromatography Workstation software (Varian Star Toolbar, version 6.8). Fibers were conditioned before each analysis by exposure within the injection port for 20 min at 250 ^o^C.

The external standard method [9,43] was used for determining concentrations of ethanol associated with the tissue core samples. Serial dilutions of ethanol ranging from 100 g/L to 0.0001 g/L were made in water and aliquots containing known amounts of ethanol were applied to filter paper discs (5 mm diam.) sealed in glass 2 mL autosampler vials for sampling purposes. Peak areas associated with the ethanol standards were measured using the Star Chromatography Workstation software. A standard concentration curve was then developed and used to determine concentrations of ethanol associated with the tissue samples.

### Preference for Attacking Tree Parts

To document species preferences for attacking certain tree parts, we compared the propensity of ambrosia beetles to attack above-ground aerial parts of flood-stressed and non-flooded *C*. *florida*. Trees were flood-stressed as previously described and permission was provided by OSU to deploy trees in a woodlot in Ohio (40°45’41.97”N 81°51’20.89”W). Flooded and non-flooded trees were arranged 1 m apart in pairs with 5 m between adjacent pairs. Six pairs of replicated trees were deployed and trees were held under field conditions for 15 days from 7 July 2014 to 22 July 2014. Attacked trees were then excised at the base and transferred to a walk-in refrigerator held at 5°C. Tunnels and galleries within trees were then dissected to recover foundress ambrosia beetles, which were quantified and identified to species. Presence of the white ambrosial form (i.e. conidia and sprout cells) of the symbiotic fungus [55] and ambrosia beetle eggs within the galleries were also recorded.

### Colonization Success on Flood-Stressed and Non-Flooded Trees

No-choice experiments were conducted in Ohio using *X*. *germanus* and in Virginia using *X*. *crassiusculus* to compare colonization success on flood-stressed and non-flooded *C*. *florida*. Bottle traps described by Ranger et al. [28] were used for obtaining field-collected adults, but instead of propylene glycol, a moistened paper towel rolled into a tube was placed in the bottom collection vessel of the trap to maintain ambrosia beetle specimens during 24 hrs under field conditions. Ambrosia beetles were then returned to the laboratory and transferred to parafilm-sealed petri dishes containing moistened filter paper and stored for 24 to 48 hrs at 3.3°C.

**Fig 1:**
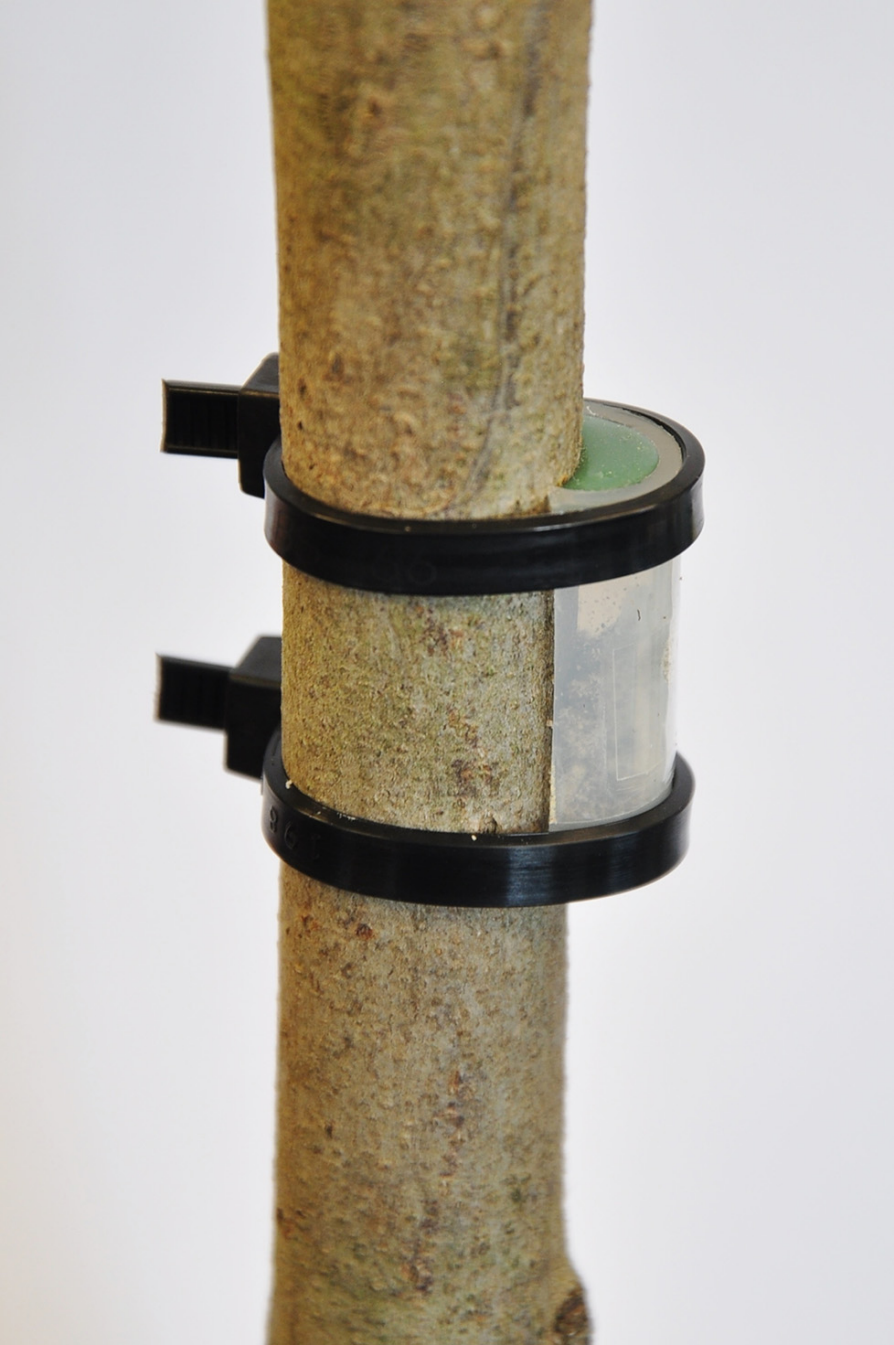
Chamber designed for confining adult female *X*. *germanus* and *X*. *crassiusculus* to stems of flood-stressed and non-flooded *Cornus florida*.

Flooding of container grown trees was established using the previously described pot-in-pot system on 19 May 2014 in a greenhouse on the campus of the OARDC and on 12 May 2014 on the campus of the HRAREC. Three days after initiating flooding, an individual adult female *X*. *germanus* field-collected in Ohio or *X*. *crassiusculus* field-collected in Virginia were placed inside of a chamber made of polytetrafluoroethylene (PTFE) tubing that was cut longitudinally (2.5 cm × 1 cm × 0.9 cm; l × w × h) and sealed at both ends with Molded Thermogreen LB-2 Septa (Supelco, Bellefonte, PA) cut into a semi-circle (Fig 1). Cables ties were used to snugly secure the chambers against the stem in parallel, thereby confining an individual beetle to 2.5 cm^2^ of bark tissue. Smooth bark of the *C*. *florida* trees allowed for close contact between the tissue and chambers, thereby effectively confining the beetle specimens. Ten chambers per tree were placed on six flood-stressed and six non-flooded *C*. *florida* trees starting from the base and extending linearly about 60 cm up the main stem with about 1.5−2 cm between cages. Generally, one to two unresponsive or injured beetles per tree were removed and replaced during the first day. Chambers confining foundress ambrosia beetles were held in place for 25 days, after which the stems were cut at the base and temporarily stored at 5°C until further analysis. As an indication of tunneling activity, chambers were carefully removed from the stems 1‒2 d later and ejected sawdust within each cage was weighed. Stem sections associated with each chamber as part of the experiment conducted in Ohio were also dissected to determine if the foundress was still alive and assess the presence/absence of eggs, larvae, pupae, and fungal growth within each tunnel/gallery. Flooded trees were drained 15 days into the experiment to avoid tree death and then watered accordingly for the remaining 10 days.

### Statistical Analyses

Cumulative ambrosia beetle attacks on trees varying in their tolerance of flooding were first examined using a repeated measures ANOVA to test for between-subject effects [56]. When a significant between-subject treatment × time effect was detected (*P* <0.05), the number of attacks per treatment at a given time point were analyzed by two-way ANOVA using tree species and flood level as main effects and tree species × flood level as an interaction [56]. Differences among treatments were determined using least-squares means (α = 0.05). Two-way ANOVA and least-squares means were also used to compare ethanol concentrations associated with the flood-stressed and non-flooded tree species (main effects = tree species and flood level; interaction = tree species × flood level). Regression analysis was used to test for a correlation between day 3 ethanol concentrations and cumulative attacks per tree associated with flood-stressed and non-flooded trees. Two-way ANOVA and least-squares means were used to compare cumulative attacks and ambrosia beetle specimens excavated per tree from stems and branches of flood-stressed and non-flooded trees (main effects = tree part and flood level; interaction = tree part × flood level). An unpaired t-test (α = 0.05) was used to compare ejected sawdust, and the percentage of galleries per tree with living foundresses, eggs, larvae, pupae, and fungal growth between flood-stressed and non-flooded trees. All data were square root transformed prior to analysis, but untransformed data are presented.

## Results

### Preference among Trees Varying in their Tolerance of Flood Stress

**Fig 2:**
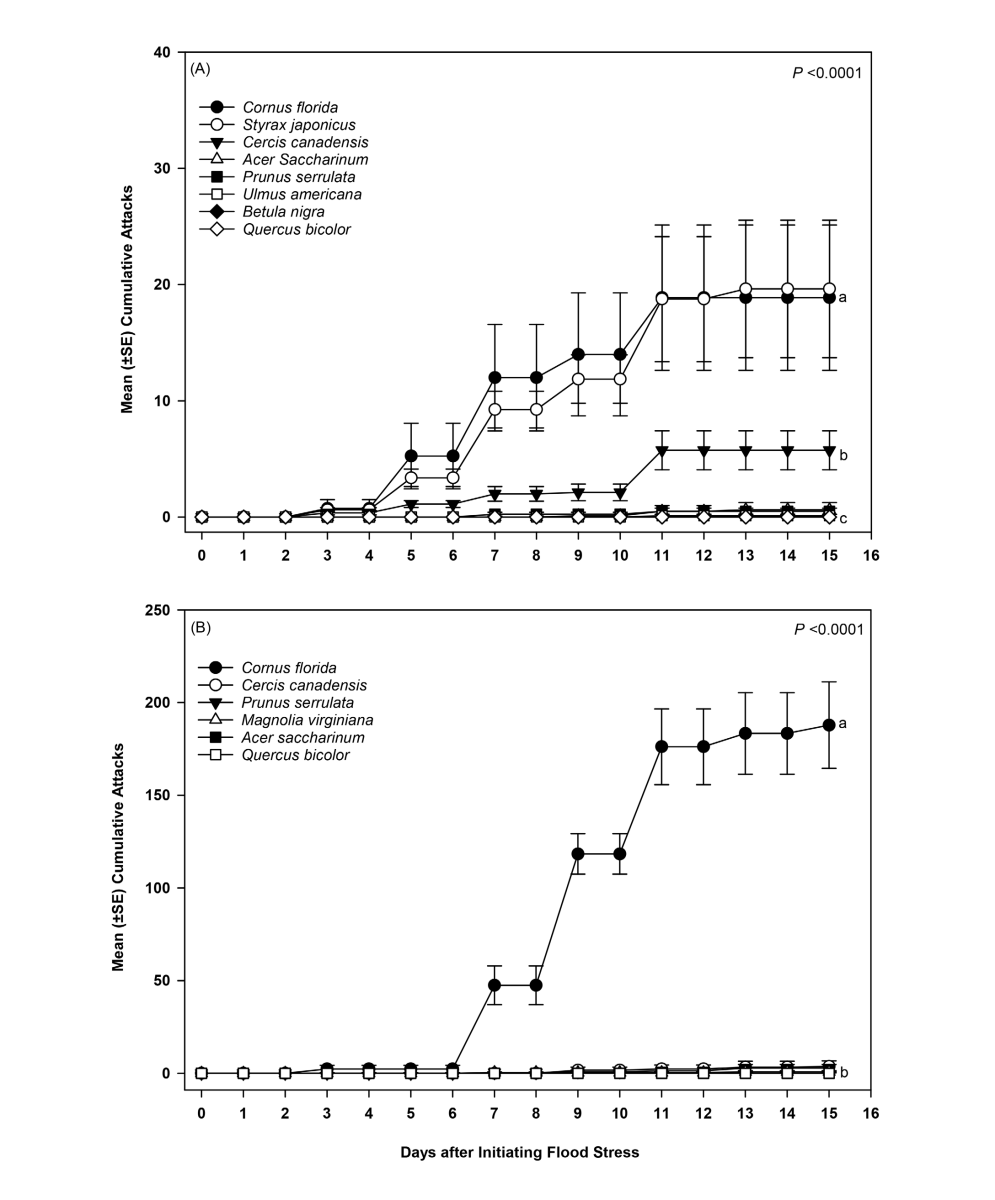
Cumulative mean (±SE) attacks by ambrosia beetles on flood-stressed trees with varying intolerance of flooding deployed in (A) Ohio and (B) Virginia. Data for non-flooded trees are not presented in the figure. Different letters indicate significant differences among treatments at day 15 by two-way ANOVA and least-squares means (*P* value provided for tree species × flooding interaction effect; see Results section for remaining output; n = 8 and n = 6 trees per treatment for Ohio and Virginia, respectively).

Within 3 days of imposing flood stress, ambrosia beetles began rapidly attacking tree species intolerant of flooding, *C*. *florida* and *S*. *japonicus*, when given free choice among flooded and non-flooded trees of varying tolerance (Fig 2A). A significant between-subject treatment × time effect was detected in cumulative ambrosia beetle attacks for trees deployed in Ohio (*F* = 22.23; df = 15,112; *P* <0.0001) (Fig 2A). By 5 days, a significant interaction effect was detected between tree species × flooding level (*F* = 12.27; df = 7; *P* <0.0001), along with significant main effects associated with tree species (*F* = 11.54; df = 7; *P* <0.0001) and flooding level (*F* = 42.18; df = 1; *P* <0.0001). Specifically, *S*. *japonicus* and *C*. *florida* sustained significantly more attacks 5 days after initiating flooding than all other flooded and non-flooded species, while *C*. *canadensis* received more attacks than all the remaining treatments. By 15 days, *C*. *florida* and *S*. *japonicus* sustained the highest number of cumulative attacks followed by *C*. *canadensis* (species × flooding interaction: *F* = 15.07; df = 7; *P* <0.0001; tree species effect: *F* = 14.59; df = 7; *P* <0.0001; flooding effect: *F* = 71.12; df = 1; *P* <0.0001). No attacks occurred on flood-stressed *B*. *nigra* and *Q*. *bicolor* or any of the non-flooded tree species throughout the duration of the experiment.

Non-native *X*. *germanus* was the predominant species excavated from galleries created in *C*. *florida* and *S*. *japonicus* deployed in Ohio, but specimens of the non-native species *Ambrosiodmus rubricollis* (Eichhoff) and native *Monarthrum mali* (Fitch) were also recovered (Table 1). *Xylosandrus germanus* (n = 102 specimens) represented 98.0% of the total ambrosia beetles recovered from *C*. *florida*, followed by 1.0% for *A*. *rubricollis* (n = 1) and 1.0% for *M*. *mali* (n = 1). Only *X*. *germanus* was recovered from flood-stressed *S*. *japonicus* (n = 112), *C*. *canadensis* (n = 35), *P*. *serrulata* (n = 8), *A*. *saccharinum* (n = 2), and *U*. *americana* (n = 1) (Table 1).

**Table 1:** Mean (±SE) cumulative attacks and ambrosia beetle specimens recovered per flood-stressed tree deployed in Ohio.

		Scolytinae recovered per tree		
Flood-stressed tree species^a^	Cumulative Attacks	*Xylosandrus germanus*	*Ambrosiodmus rubricollis*	*Monarthrum mali*
*Styrax japonicus*	19.6 ± 5.9	14.0 ± 5.9	0.0 ± 0.0	0.0 ± 0.0
*Cornus florida*	18.9 ± 6.3	12.8 ± 4.6	0.1 ± 0.1	0.1 ± 0.1
*Cercis canadensis*	6.6 ± 1.6	4.4 ± 1.5	0.0 ± 0.0	0.0 ± 0.0
*Prunus serrulata*	1.3 ± 0.7	1.0 ± 0.6	0.0 ± 0.0	0.0 ± 0.0
*Acer saccharinum*	0.6 ± 0.4	0.3 ± 0.2	0.0 ± 0.0	0.0 ± 0.0
*Ulmus americana*	0.1 ± 0.1	0.1 ± 0.1	0.0 ± 0.0	0.0 ± 0.0
*Betula nigra*	0.0 ± 0.0	0.0 ± 0.0	0.0 ± 0.0	0.0 ± 0.0
Quercus bicolor	0.0 ± 0.0	0.0 ± 0.0	0.0 ± 0.0	0.0 ± 0.0

^a^ No attacks occurred on non-flooded trees (n = 6 trees per treatment).

A significant between-subject treatment × time effect was also detected for flood-stressed and non-flooded trees deployed in Virginia (*F* = 158.34; df = 11, 60; *P* <0.0001) (Fig 2B). Attacks began to occur on flood-stressed *C*. *florida* three days after initiating flooding. By 7 days, a significant interaction effect was detected between tree species × flooding level (*F* = 43.8; df = 5; *P* <0.0001), along with significant effects associated with the main factors of tree species (*F* = 42.8; df = 5; *P* <0.0001) and flooding level (*F* = 48.6; df = 1; *P* <0.0001). *Cornus florida* sustained more attacks 7 days after initiating flooding than all other flooded and non-flooded species.

By 15 days after initiating flooding, more cumulative attacks occurred on flood-stressed *C*. *florida* than all other flooded and non-flooded trees (species × flooding: *F* = 107.1; df = 5; *P* <0.0001; tree species effect: *F* = 106.3; df = 5; *P* <0.0001; flooding effect: *F* = 162.2; df = 1; *P* <0.0001). Considerably fewer attacks occurred on flood-stressed *C*. *canadensis* and *P*. *serrulata*, which were higher than all the remaining non-flooded and flooded species treatments except flood-stressed *M*. *virginiana*. No difference in attacks were detected among non-flooded *A*. *saccharinum*, *C*. *canadensis*, *C*. *florida*, *M*. *virginiana*, *P*. *serrulata*, and *Q*. *bicolor*, along with flood-stressed *A*. *saccharinum*, *M*. *virginiana*, and Q. *bicolor*. No attacks occurred on flood-stressed *A*. *saccharinum* and *Q*. *bicolor*, or non-flooded *C*. *florida*, *M*. *virginiana*, and *P*. *serrulata*. However, one non-flooded *C*. *canadensis* sustained one attack.

Only non-native ambrosia beetles attacked experimental trees deployed in Virginia. *Xylosandrus germanus* was the predominant species excavated from galleries created in *C*. *florida* deployed in Virginia, but specimens of non-native *X*. *crassiusculus* and non-native *Xylosandrus compactus* (Eichhoff) were also recovered (Table 2). *Xylosandrus germanus* (n = 241 specimens) represented 70.3% of the total ambrosia beetles recovered from *C*. *florida*, followed by 28.6% for *X*. *crassiusculus* (n = 98) and 1.2% for *X*. *compactus* (n = 4).

**Table 2:** Mean (±SE) cumulative attacks and ambrosia beetle specimens recovered per flood-stressed tree deployed in Virginia.

		Scolytinae recovered per tree		
Flood-stressed tree species ^a^	Cumulative Attacks	*Xylosandrus germanus*	*Xylosandrus crassiusculus*	*Xylosandrus compactus*
*Cornus florida*	187.8 ± 23.3	40.2 ± 7.0	16.3 ± 7.3	0.7 ± 0.7
*Cercis canadensis*	3.7 ± 3.1	1.0 ± 0.8	0.0 ± 0.0	0.0 ± 0.0
*Prunus serrulata*	2.8 ± 1.9	0.0 ± 0.0	0.0 ± 0.0	0.0 ± 0.0
*Magnolia virginiana*	0.8 ± 0.7	0.0 ± 0.0	0.0 ± 0.0	0.0 ± 0.0
*Acer saccharinum*	0.0 ± 0.0	0.0 ± 0.0	0.0 ± 0.0	0.0 ± 0.0
*Quercus bicolor*	0.0 ± 0.0	0.0 ± 0.0	0.0 ± 0.0	0.0 ± 0.0

^a^ No attacks occurred on the non-flooded trees, except for 0.2 ± 0.2 attacks on non-flooded *C*. *canadensis* (see Results section; n = 6 trees per treatment).

### Association of Ethanol with Trees Varying in their Tolerance of Flood Stress

**Fig 3:**
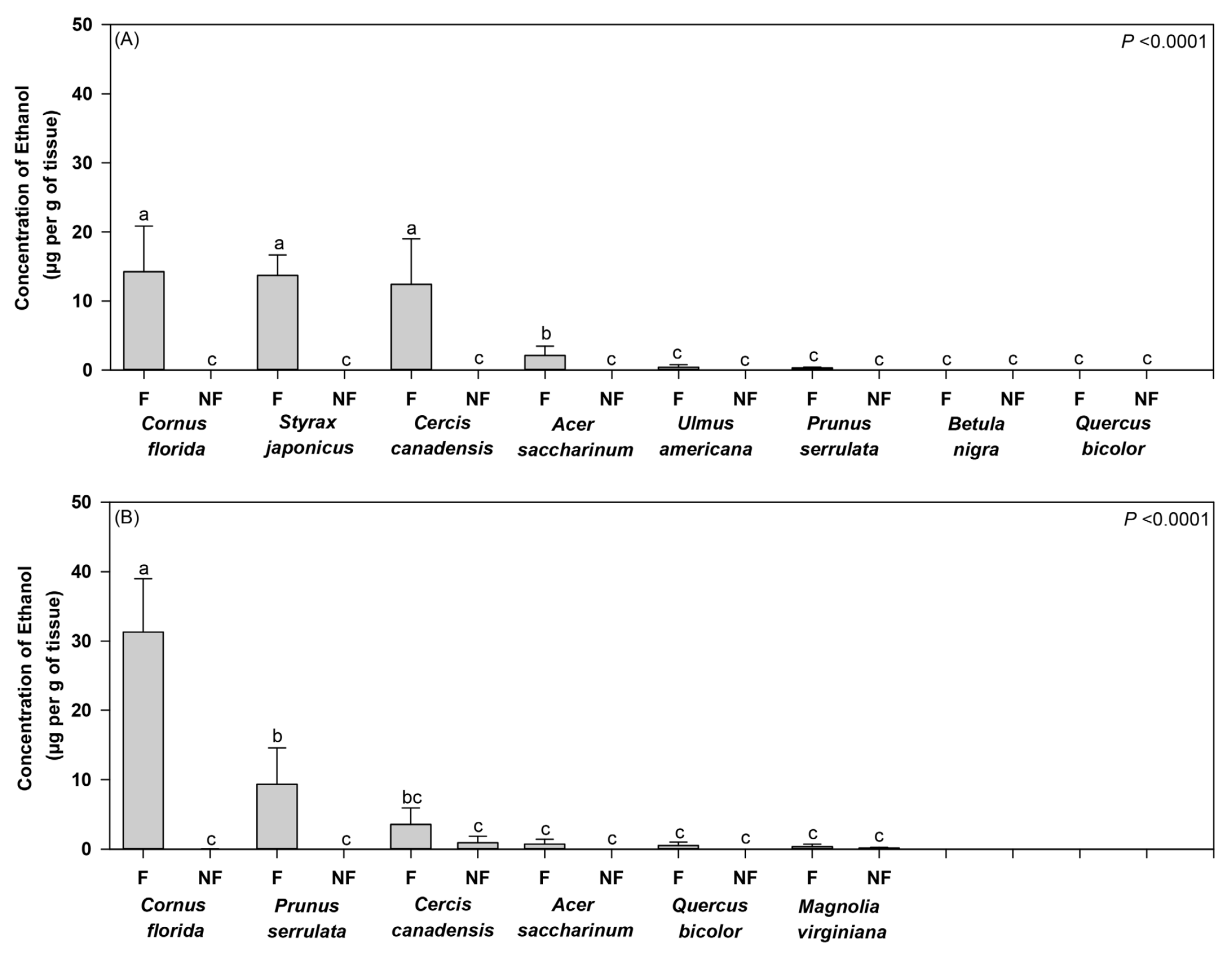
Mean (±SE) ethanol concentrations associated with flood-stressed and non-flooded trees of varying intolerance to flooding deployed in (A) Ohio and (B) Virginia. Ethanol concentrations were analyzed 3 days after initiating flood stress. Different letters indicate significant differences among treatments by two-way ANOVA and least-squares means (*P* value provided for tree species × flooding interaction effect; see Results section for remaining output; n = 8 and n = 6 trees per treatment for Ohio and Virginia, respectively).

**Fig 4:**
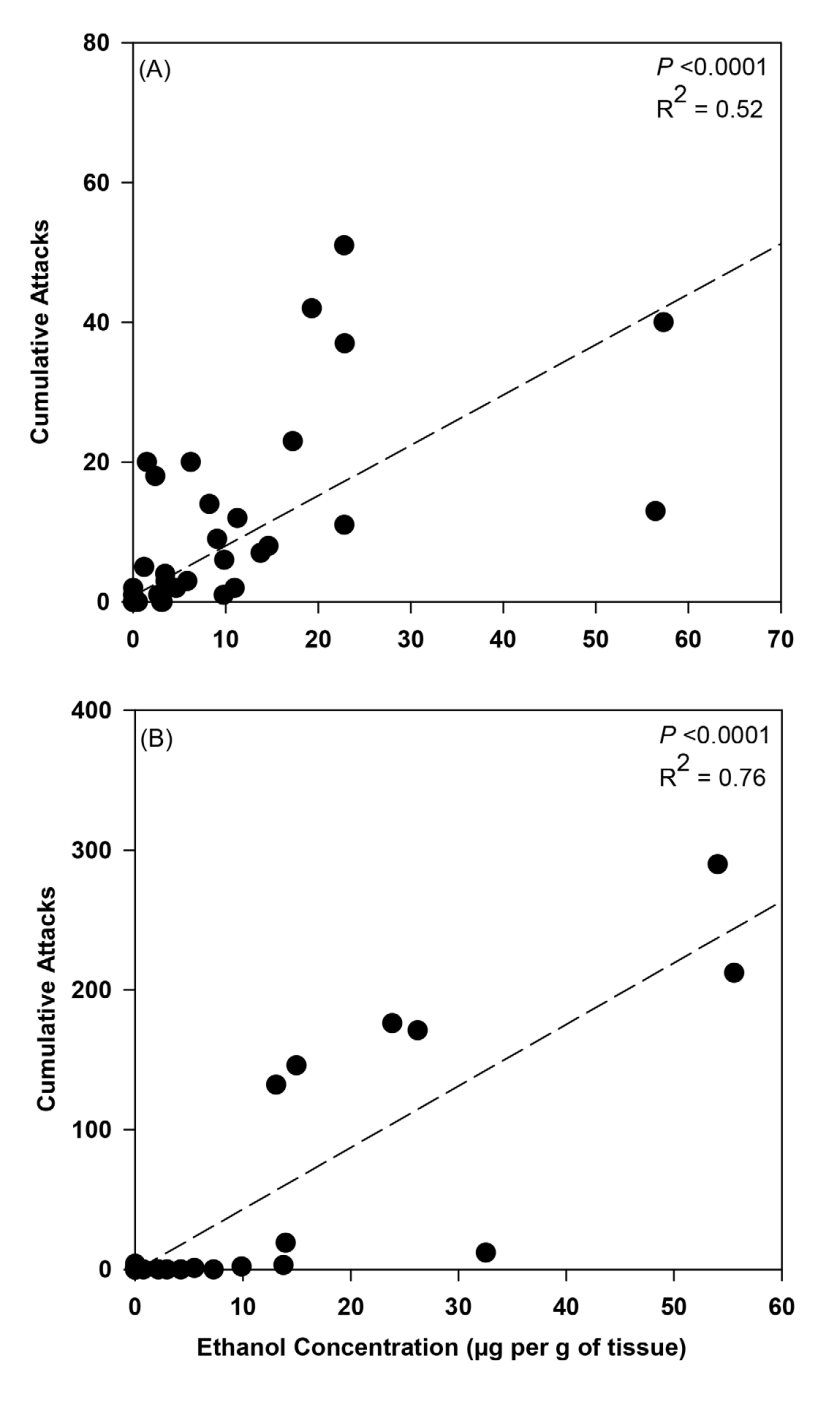
Correlation between day 3 ethanol concentrations and cumulative ambrosia beetles attacks per flood-stressed and non-flooded tree deployed in (A) Ohio and (B) Virginia. See Fig 3A and 3B for tree species tested and Results section for additional statistical output (n = 128 and n = 72 trees deployed in Ohio and Virginia, respectively).

At 3 days after initiating flooding, differences were detected in ethanol concentrations associated with trees varying in their tolerance of flooding. A significant interaction effect on ethanol concentrations was detected between tree species and flooding for trees deployed in Ohio (*F* = 10.29; df = 7; *P* <0.0001) (Fig 3A). Significant main effects were also associated with tree species (*F* = 9.85; df = 7; *P* <0.0001) and flood stress (*F* = 59.41; df = 1; *P* <0.0001). Higher levels of ethanol were detected in tissues collected from flood-stressed *C*. *florida*, *S*. *japonicus*, and *C*. *canadensis* compared to all the remaining treatments (Fig 3A). Ethanol levels associated with *A*. *saccharinum* were higher than the remaining treatments, while no differences were detected among flooded and non-flooded *Q*. *bicolor*, *B*. *nigra*, *U*. *americana*, and *P*. *serrulata*, and non-flooded *A*. *saccharinum*, *C*. *canadensis*, *C*. *florida*, and *S*. *japonicus*. A significant positive correlation (r^2^ = 0.52) was detected between day 3 ethanol concentrations and cumulative attacks on the flood-stressed and non-flooded trees (*F* = 129.03; df = 1, 126; *P* <0.0001) (Fig 4A).

A significant interaction was also detected between tree species and flooding level on ethanol concentrations associated with trees deployed in Virginia (*F* = 12.30; df = 5; *P* <0.0001), along with significant main effects for tree species (*F* = 9.94; df = 5; *P* <0.0001) and flooding level (*F* = 32.92; df = 1; *P* <0.0001) (Fig 3B). Higher levels of ethanol were detected in flood-stressed *C*. *florida* tissues compared to all the remaining flooded and non-flooded treatments (Fig 3B). Ethanol levels associated with flood-stressed *P*. *serrulata* were considerably lower than *C*. *florida*, but higher than all the remaining treatments except for *C*. *canadensis*. A significant positive correlation (r^2^ = 0.76) was detected between day 3 ethanol concentrations and cumulative attacks on the flood-stressed and non-flooded trees (*F* = 222.61; df = 1, 70; *P* <0.0001) (Fig 4B).

### Preference for Attacking Tree Parts

**Fig 5:**
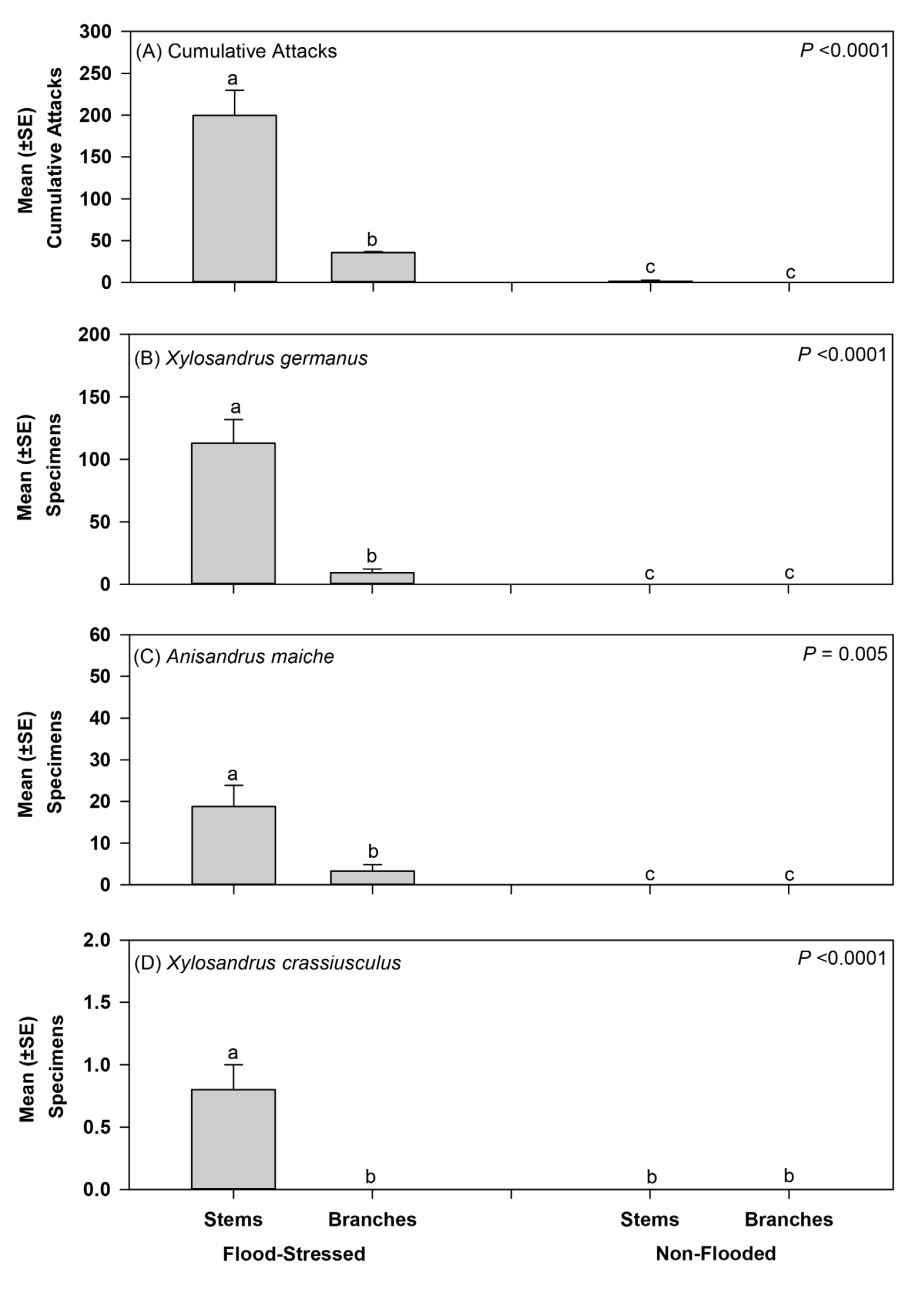
Mean (±SE) cumulative attacks and specimens excavated per tree from stems and branches of flood-stressed and non-flooded *Cornus florida*. Different letters within a biological parameter indicate significant differences by two-way ANOVA and least-squares means (*P* value provided for tree part × flooding interaction effect; see Results section for remaining output; n = 6 flood-stressed and non-flooded trees).

Stems of flood-stressed *C*. *florida* deployed under field conditions in Ohio were preferentially attacked over branches of flood-stressed trees, and stems and branches of non-flooded trees (Fig 5A–5D). A mean of 199.8 and 35.7 attacks occurred on stems and branches of flood-stressed trees, while 1.5 and 0.0 attacks occurred on stems and branches of non-flooded trees, respectively (Fig 5A). A significant interaction was detected between the influence of tree part and incidence of flood stress on cumulative attacks (*F* = 25.18; df = 1; *P* <0.0001), along with significant main effects associated with tree part (*F* = 34.08; df = 1; *P* <0.0001) and flood stress (*F* = 145.70; df = 1; *P* <0.0001) (Fig 5A). Attacks were not observed to occur on any tree parts other than stems and branches.

Stems on two of the six non-flooded *C*. *florida* trees sustained one and eight cumulative attacks per tree; no attacks occurred on the remaining non-flooded trees. The two non-flooded trees attacked by ambrosia beetles were paired with the two most heavily attacked flood-stressed trees that sustained 338 and 337 attacks per tree, respectively, compared to the remaining flood-stressed trees with 173, 260, 120, and 185 attacks per tree. Only superficial tunneling occurred on the two non-flooded trees and no ambrosia beetles were excavated from the tunnels (Fig 5B–5D). In contrast, a mean (±SE) of 23.0 ± 5.2 percent of attacks on the flood-stressed trees resulted in galleries, 73.3 ± 6.4 percent of which contained the white ambrosial form (i.e. conidia and sprout cells) of the symbiotic fungus and 14.0 ± 3.9 percent of which contained symbiotic fungus and ambrosia beetle eggs, respectively.

Only non-native ambrosia beetles were excavated from the flood-stressed trees (Fig 5A–5D). *Xylosandrus germanus* (n = 676) represented 85.1% of the total specimens excavated from the stems, followed by 14.2% for *Anisandrus maiche* Stark (n = 113) and 0.6% for *X*. *crassiusculus* (n = 5). *Xylosandrus germanus* (n = 56) was also the dominant species recovered from *C*. *florida* branches and represented 73.7% of the total excavated specimens followed by 26.3% for *A*. *maiche* (n = 20).

More *X*. *germanus*, *A*. *maiche*, and *X*. *crassiusculus* were excavated from stems of flood-stressed trees compared to branches on flood-stressed trees and stems and branches on non-flooded trees (Fig 5B–5D). A significant interaction was detected between the influence of tree part and flood stress on the number of *X*. *germanus* excavated per tree (*F* = 162.10; df = 1; *P* <0.0001), along with significant main effects for tree part (*F* = 52.72; df = 1; *P* <0.0001) and impact of flood stress (F = 54.24; df = 1; P < 0.0001) (Fig 5B). A significant interaction (*F* = 9.91; df = 1; *P* <0.0001), tree part main effect (*F* = 9.15; df = 1; *P* = 0.007), and flood stress main effect (*F* = 40.66; df = 1; *P* < 0.0001) were detected for *A*. *maiche* (Fig 5C). A significant interaction (*F* = 25.01; df = 1; *P* <0.0001), tree part main effect (*F* = 25.01; df = 1; *P* <0.0001), and flood stress main effect (*F* = 25.01; df = 1; *P* <0.0001) were also detected for *X*. *crassiusculus* (Fig 5D).

### Colonization Success on Flood-Stressed and Non-Flooded Trees

**Fig 6:**
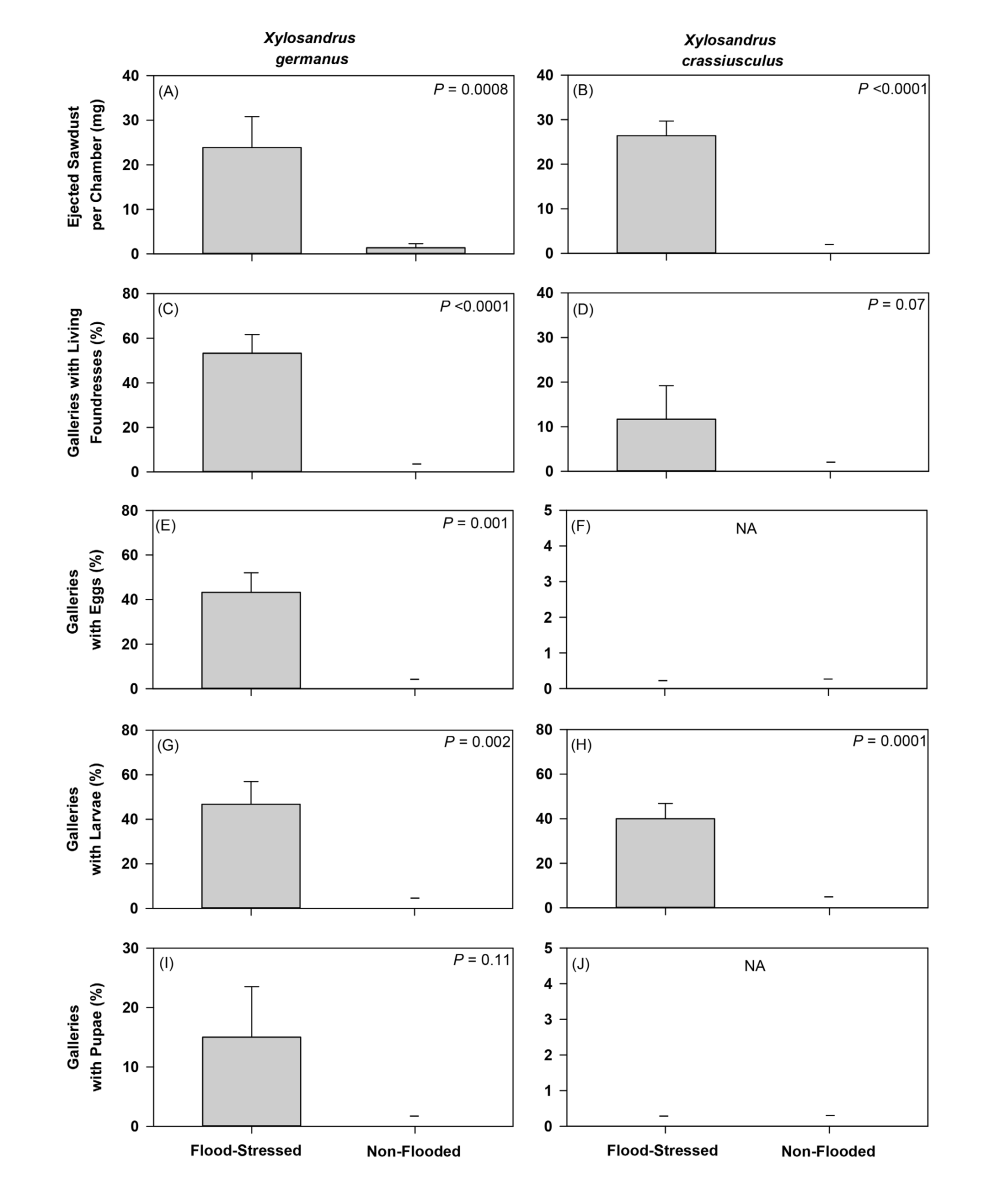
Tunneling activity and colonization success of *Xylosandrus germanus* and *Xylosandrus crassiusculus* under no-choice conditions on flood-stressed and non-flooded *Cornus florida*. Mean (±SE) ejected sawdust per chamber (A-B) and percentage of galleries per tree with living foundresses (C-D), eggs (E-F), larvae (G-H), and pupae (I-J). Tunnels/galleries were excavated 25 days after confining individual foundress beetles. Differences within a species and biological parameter were compared using an unpaired t-test (see Results section for full statistical analysis output; 10 chambers containing 1 adult female were placed on n = 6 flood-stressed and non-flooded trees; NA = no analysis).

When confined under no-choice conditions, more sawdust resulting from burrowing/tunneling activities accumulated in chambers confining adult female *X*. *germanus* (*t* = 4.74; df = 10; *P* = 0.0008) and *X*. *crassiusculus* (*t* = 7.91; df = 10; *P* <0.0001) to flood-stressed *C*. *florida* compared to non-flooded trees (Fig 6A and 6B). Living foundress *X*. *germanus* were detected within tunnels/galleries created in flood-stressed *C*. *florida*, but no living specimens were associated with non-flooded trees (*t* = 11.38; df = 10; *P* <0.0001) (Fig 6C). Living foundress *X*. *crassiusculus* were also detected within tunnels/galleries created in flood-stressed *C*. *florida*, but no living specimens were associated with non-flooded trees (*t* = 1.58; df = 10; *P* = 0.07) (Fig 6D). Eggs of *X*. *germanus* were detected in galleries created in flood-stressed trees, but no eggs were associated with beetles confined to non-flooded trees (*t* = 4.65; df = 10; *P* = 0.001) (Fig 6E). No *X*. *crassiusculus* eggs were associated with flood-stressed or non-flooded trees (Fig 6F). Larvae of *X*. *germanus* (*t* = 4.17; df = 10; *P* = 0.002) and *X*. *crassiusculus* (*t* = 4.17; df = 10; *P* = 0.002) were recovered from galleries within flood-stressed trees, but no larvae were associated with non-flooded trees (Fig 6G and 6H). Pupae of *X*. *germanus* were recovered from flood-stressed trees, but not from non-flooded trees (*t* = 0.11; df = 10; *P* = 0.11) (Fig 6I). No *X*. *crassiusculus* pupae were associated with flood-stressed or non-flooded trees (Fig 6J).

## Discussion

Ambrosia beetles, including *X*. *germanus* and *X*. *crassiusculus*, have traditionally been viewed as secondary colonizers of dying or dead trees, or those already under attack by more aggressive tree killing bark beetles [4,6]. Attacks on living trees, even within non-native habitats, were limited to injured parts of trees [27]. Yet, a variety of ambrosia beetles established in non-native habitats have reportedly begun attacking living trees [4,6,27]. Results from our current study demonstrate that while living trees were indeed attacked by non-native *X*. *germanus* and *X*. *crassiusculus*, beetles efficiently distinguished among varying host qualities and rapidly targeted species intolerant of flood stress. A few attacks occurred on non-flooded trees under free choice field conditions, but the tunnels were superficial, abandoned, and no galleries were formed. Subsequent experiments under no-choice conditions demonstrated that both *X*. *germanus* and *X*. *crassiusculus* readily colonized flood-stressed trees, as indicated by the production of eggs, larvae, and pupae, but were unwilling or unable to colonize non-flooded trees. A positive correlation was also demonstrated between ethanol stem concentrations and ambrosia beetle attacks. We therefore propose the basis for the shift by *X*. *germanus* and *X*. *crassiusculus* from attacking dying/dead trees to living ones involves early colonization of living but weakened trees.

Hulcr and Dunn [4] suggested a component of exotic ambrosia beetles attacking living trees may involve an ‘olfactory mismatch’ whereby beetles perceive volatiles emitted from live trees as representative of emissions from dead trees. Our current study indicates the stress-induced emission of ethanol from living trees plays an important role in the selection of living but weakened trees. An asymptomatic outward appearance of living but weakened trees in the early onset of physiological stress can be anthropogenically perceived to indicate a living and healthy tree being targeted for attack [43]. However, physiologically stressed trees can visually appear healthy, but emit ethanol and other stress-related compounds that function as important host-location cues for opportunistic ambrosia beetles [9,43]. Ethanol was detected within stem tissues of flood-stressed trees by 3 days after initiating flooding as part of our current study. Beetles also began attacking flood-stressed *C*. *florida* and *S*. *japonicus* within 3 days of initiating flooding as part of our current study. Similarly, *X*. *germanus* began landing on flood-stressed *C*. *florida* within 1 day of initiating flooding [43]. These results indicate the rapid production and emission of stress-induced volatiles, particularly ethanol, from otherwise apparently-healthy trees are advantageously used by *X*. *germanus* and *X*. *crassiusculus* to locate vulnerable hosts. Rapidly locating resources in a weakened physiological state that are often spatially and temporally unpredictable is an important adaptive trait for insects that primarily or exclusively target stressed hosts [57]. Thus, selection pressures may be favoring individuals that rapidly locate and successfully colonize living but recently weakened trees.

Semiochemical-mediated attacks by aggressive bark beetles on an individual tree are demonstrated to result in beetles ‘spilling over’ and attacking neighboring healthy trees [58,59]. Such spill-over of *X*. *germanus* and *X*. *crassiusculus* from heavily infested trees onto neighboring healthy trees may occur under natural settings, and colonization may then be facilitated by infecting the hosts with symbiotic and auxiliary fungi. Spill-over could explain the clustering of attacked trees documented to occur in diverse landscapes [9], but localized site conditions may have predisposed clusters of trees to attack. However, numerous free-choice experiments have not detected attacks by *X*. *germanus*, *X*. *crassiusculus*, and other ambrosia beetles on untreated trees adjacent to flood-stressed trees or those baited, irrigated, or injected with ethanol [9,28,29,31–33,60]. A few attacks on neighboring non-flooded (and apparently-healthy) trees were documented as part of our current study, but the tunnels were superficial and abandoned. If *X*. *germanus* and *X*. *crassiusculus* do attempt to colonize healthy trees adjacent to stressed ones, our current study suggests they are poor colonizers of such trees. Since oviposition by various Xyleborine species does not occur prior to establishment of the symbiotic fungus [61], host factors disrupting fungal establishment could have important implications for the role of host physiological condition on tree utilization by non-native species.

Our current study used flood-stress as a technique for characterizing attacks on living trees by *X*. *germanus* and *X*. *crassiusculus*, particularly the role of host physiological condition on preference behavior and colonization success. Other physiological stressors also likely play a role in beetles targeting living but weakened trees in natural and managed non-native habitats. For instance, spring frost events following a record-setting mild winter preceded attacks on trees intolerant of late frost injury, namely, *Acer palmatum* Thunb., *C*. *canadensis*, *Liridodendron tulipifera* L., *S*. *japonicus*, and *Zelkova serrata* (Thunb.) [9]. Similarly, forested stands of beech trees, *Fagus sylvatica* L., regarded as apparently-healthy at the time of attack by *X*. *germanus* and other ambrosia beetles were subjected to extreme frost events in the year prior to initiation of wide spread attacks [11,62]. Subsequent studies [62] demonstrated experimentally frost injured tissues on *F*. *sylvatica* trees were more attractive than non-injured trees to *X*. *germanus* and other non-native ambrosia beetles. Ambrosia beetle attacks were also observed on the frost injured tissues.

Generally, researchers rarely have the opportunity to assess the physiological condition of a host prior to discovering the initiation of attack by wood-boring insects [63]. As a result, many species initially considered fully capable of colonizing trees ranging from healthy to decaying were subsequently found to restrict their attacks to hosts in a specific condition. Ploetz et al. [6] discussed examples of ambrosia beetle attacks and associated diseases in orchards and ornamental nurseries, and pointed out that in none of the provided cases was the pre-existing health of the trees known at the time of attack. However, the pronounced incidences of extensive attacks and associated diseases within the orchards/nurseries compared to natural settings suggests the affected trees were predisposed to attack [6]. Similarly, field observations involving orchard-grown black walnut trees, *Juglans nigra* L., considered apparently-healthy at the time of attack were serendipitously determined to have exhibited slower growth rates in the year before attack compared to non-attacked trees [21]. Dieback, basal sprouts, and *Fusarium* cankers were also associated with trees not attacked by *X*. *germanus* [22], thereby indicating trees within the orchard may have been in a weakened state prior to beetles attacking.

Physiological stressors that initially predispose living but weakened trees to attack by ambrosia beetles could also predispose the trees to infection by the symbiotic and auxiliary fungi after attacks are initiated [6,64]. Trees could conceivably become even more attractive due to the emission of ethanol and other key volatiles from cankerous tissue and wilting foliage following infection. However, the role of symbiotic and auxiliary fungi on host interactions and colonization success of *X*. *germanus*, *X*. *crassiusculus*, and numerous other non-native ambrosia beetles is not well characterized [6]. *Ambrosiella hartigii* Batra is reportedly the symbiotic fungus of *X*. *germanus*, but *Cerarocystis ulmi* (Buisman), *Fusarium lateritium* Nees, *Fusarium solani*, and *Fusarium oxysporum* Schlechter ex Fries have been isolated from their galleries and cankers [65–69]. *Ambrosiella roeperi* sp. nov. has recently been identified as the mycangial symbiont of *X*. *crassiusculus* [70].

In addition to preferentially attacking flood-stressed trees, results from our current study also documented a preference by *X*. *germanus* and *X*. *crassiusculus* for attacking stems over branches on flood-stressed *C*. *florida*. Our current study also appears to be the first recovery of *A*. *maiche* from a host tree in North America [71] and demonstrated stems over branches on living but weakened trees were preferentially attacked. Similarly, more than 90% of ambrosia beetle attacks occurred on the main stem of *C*. *florida* trees growing at an ornamental nursery [72], and the majority of attacks by *X*. *germanus* and *X*. *crassiusculus* occurred less than 30 cm above the ground on stems of chestnut *Castanea mollissima* Blume [25]. Beetle preferences for certain tree parts may be due to variations in anatomy and physiology between stems and branches [73,74] or represent a strategy to minimize interspecific competition among ambrosia beetles. Attacks on stems could have a more deleterious impact on whole-tree physiology than attacks on the branches due to disruption of the transpiration stream within xylem vessels by symbiotic or auxiliary fungal colonization and/or host hypersensitive responses to infection.

Although not always consistent [36], higher amounts of ethanol have been detected from flood stress intolerant tree species compared to tolerant species [44,45]. *Cornus florida* is generally recognized as intolerant of flooding [49,51], and flood-stressed *C*. *florida* deployed in Ohio and Virginia were associated with the highest concentrations of ethanol and cumulative ambrosia beetles as part of their respective experiments. However, flood-stressed *C*. *canadensis* deployed in Ohio and *P*. *serrulata* deployed in Virginia were associated with relatively high ethanol concentrations, but few ambrosia beetle attacks. This discrepancy may be attributed to interspecific variability in metabolic responses [37,75] and suggests vascular tissue concentrations of ethanol may not always reflect emission rates from the epidermis. Monitoring the emission of ethanol and other stress-induced volatiles from the epidermis using SPME-GC-MS [43] may be an improved approach for characterizing the role of host-derived volatiles during host selection by opportunistic ambrosia beetles. Differences in tissue processing/handling and sampling locations, and personnel may have resulted in intraspecific variability in ethanol concentrations, particularly for *C*. *florida*, *C*. *canadensis*, and *P*. *serrulata*. However, we limited our comparisons of ethanol concentrations among trees to only those deployed within a specific location for the purposes of our current study.

Overall, our current study indicates *X*. *germanus* and *X*. *crassiusculus* established in non-native habitats are early colonizers of living but weakened trees. This strategy may be contributing to their invasion success and proliferation. Healthy hosts may be opportunistically attacked by *X*. *germanus* and *X*. *crassiusculus*, but with limited success at colonization. Our current study also indicates the non-native *A*. *maiche* preferentially attacks living but weakened trees. Thus, other non-native ambrosia beetles that use stress-induced ethanol for locating vulnerable trees, and exhibit a capability for attacking a wide range of tree genera, may use a similar strategy.

## Supporting Information

S1 Fig Supporting data set for Fig 2A–2B. (XLSX)

S1 Table Supporting data set for Table 1. (XLSX)

S2 Table Supporting data set for Table 2. (XLSX)

S2 Fig Supporting data set for Fig 3A–3B. (XLSX)

S3 Fig Supporting data set for Fig 4A–4B. (XLSX)

S4 Fig Supporting data set for Fig 5A–5D. (XLSX)

S5 Fig Supporting data set for Fig 6A–6J. (XLSX)
